# ﻿Lateral and longitudinal dispersal of aquatic insects in mountain streams, with notes about Trichoptera

**DOI:** 10.3897/zookeys.1263.150229

**Published:** 2025-12-10

**Authors:** Tatiana Latorre-Beltrán, Ivan Arismendi, Blanca Rios-Touma, William Joseph Gerth, Alexander Petty

**Affiliations:** 1 Department of Fisheries, Wildlife, and Conservation Sciences, Oregon State University, Corvallis, Oregon, USA; 2 Ingenieria Ambiental- Facultad de Ingenierias y Ciencias Aplicadas, Universidad de las Americas, BIOMAS, Quito, Ecuador; 3 Colegio de Ciencias Biologicas y Ambientales, Universidad San Francisco de Quito, Quito, Ecuador; 4 School of Electrical Engineering and Computer Science, Oregon State University, Corvallis, Oregon, USA

**Keywords:** Aquatic subsidy, caddisflies, Malaise trap, riparian microclimate, sticky trap

## Abstract

Understanding the spatial patterns of energy flow from mountain streams via emerging aquatic insects remains limited due to a lack of empirical data. Upon emergence, adult flying insects disperse in both longitudinal (upstream/downstream) and lateral (into terrestrial habitats) directions. Here, we quantified the dispersal patterns of adult aquatic insects in both dimensions using a combination of sticky and Malaise traps. To assess longitudinal dispersal, we deployed sticky traps in transects along three streams, with Petri dish arrays designed to capture insects flying upstream, downstream, or laterally across the channel. Lateral dispersal was measured using the same trap design placed at increasing distances (up to 32–64 m) from the stream edge, complemented by Malaise traps at one site. Trichoptera exhibited the highest family-level richness among captured taxa, and the genus *Micrasema* showed a clear exponential decay in abundance with distance from the stream, consistent with lateral dispersal theory. Our findings contribute empirical evidence on the spatial extent of aquatic insect emergence and dispersal, informing future studies on stream–riparian energy flow across larger spatial and temporal scales.

## ﻿Introduction

After their immature life stages in the water, aquatic insect species emerge to complete their life cycles (Merritt et al. 2019). Winged species of aquatic insects use permanent movement to travel from the water to settlement at a new place, a phenomenon known as dispersal (Lowe and McPeek 2014). Understanding how aquatic populations and communities in headwaters disperse involves population genetics (Finn et al. 2007), colonization of new sites, and diversification of already diverse groups such as Trichoptera (Lancaster et al. 2024b). To date, most of the hypotheses related to dispersal in adult aquatic insects are supported by little empirical evidence (Lancaster et al. 2024b).

Winged adults of aquatic insects move in different dimensions: lateral, longitudinal, and vertical. Identifying the distances covered by species in these dimensions improves the understanding of the effect of land use on ecological and evolutionary processes (Didham et al. 2012) as well as helps to explain the life history traits of aquatic species using terrestrial systems (Collier and Smith 2000; Rahman et al. 2021). A recent meta-analysis compiled empirical studies on distances traveled of 180 European aquatic species to build a Dispersion Index (Peredo Arce et al. 2021) and found species capable of flight up to 20 km, such as the mayfly, *Ephoron virgo*. However, most species remain close to the water source and abundances correlate negatively with distance under different models as negative power function, negative exponential, and negative linear function (Muehlbauer et al. 2014). The lateral dispersal of Trichoptera confirms the pattern of negative correlation with the distance from the stream to the riparian zone in different regions such as in Brazil, where a steep drop was observed from 30 m (Pereira et al. 2024), or in Denmark where the Glossosomatidae family reached 20 m (Sode and Wiberg‐Larsen 1993) or in Wales, where most of the caddisfly diversity remained within 40 m from the stream bank (Petersen et al. 2004).

Riparian vegetation can modulate the microclimate, which is defined as the set of climatic conditions such as temperature, moisture, wind speed, and light in a small area (Chen et al. 1999). The microclimate of the riparian ecotone influences different life stages of aquatic and semiaquatic species (Davies-Colley and Payne 2000). Microclimate variables can also influence the habitat structure for adult aquatic insects and their possibilities of dispersal; for example, air temperature and humidity, which change due to the type of vegetation adjacent to the streams, influence the life span of emerging insects (Briers and Gee 2004).

Some studies suggest aquatic adults in general fly more over the water (longitudinally in both directions of the upstream-downstream gradient) than laterally (Sode and Wiberg‐Larsen 1993) and may travel long distances upstream, as evidenced by a hydropsychid species (Coutant 1982). Others (Lancaster et al. 2024b) highlight the importance of topography, stream order, and connectivity to disperse. However, more studies have focused on the aquatic insects dispersing longitudinally by drifting downstream as larvae and the tendency to fly upstream (i.e., the freshwater insect colonization cycle (Müller 1982). Although empirical evidence about the ratio of male–female flying upstream is scarce, some studies suggest that more females fly upstream (e.g. Bird and Hynes 1981). Moreover, other aspects of the longitudinal dispersal of adult aquatic insects have been overlooked, as the role of microclimate variables such as wind play in the longitudinal dispersal of most aquatic insects. In a study in Wales, for example, stoneflies’ dispersal was positively related to wind speed (Briers et al. 2003).

Previous studies on the aquatic insect communities of the H.J. Andrews Experimental Forest, Oregon have documented the responses of aquatic insect emergence to forest harvest (Frady et al. 2007) and temperature, but focused on common species such as *Dolophilodes dorca* (Finn et al. 2022). Yet, questions remain about their patterns of dispersal, especially for species that have not yet been taxonomically resolved, including *Sisko* spp. In addition, it is unclear if large Trichoptera can disperse longer distances compared to smaller individuals. Here, we evaluate the lateral dispersal of adult aquatic insect genera and species from the stream channel into the riparian forest (lateral dispersal). We expect that most abundant species will display a dispersal represented by a negative decay function (Muehlbauer et al. 2014) with the majority of individuals remaining close to the stream channel. Furthermore, we present a simple procedure to assess patterns of longitudinal dispersal among adults and their association with prevailing wind patterns. We expect most of the aquatic insects that emerge will disperse in the same direction as the wind. Our study provides baseline information for future work on the dispersal of adult aquatic insects after land use changes such as forest harvest and wildfires.

## ﻿Methods

### ﻿Study site

**Figure 1.**
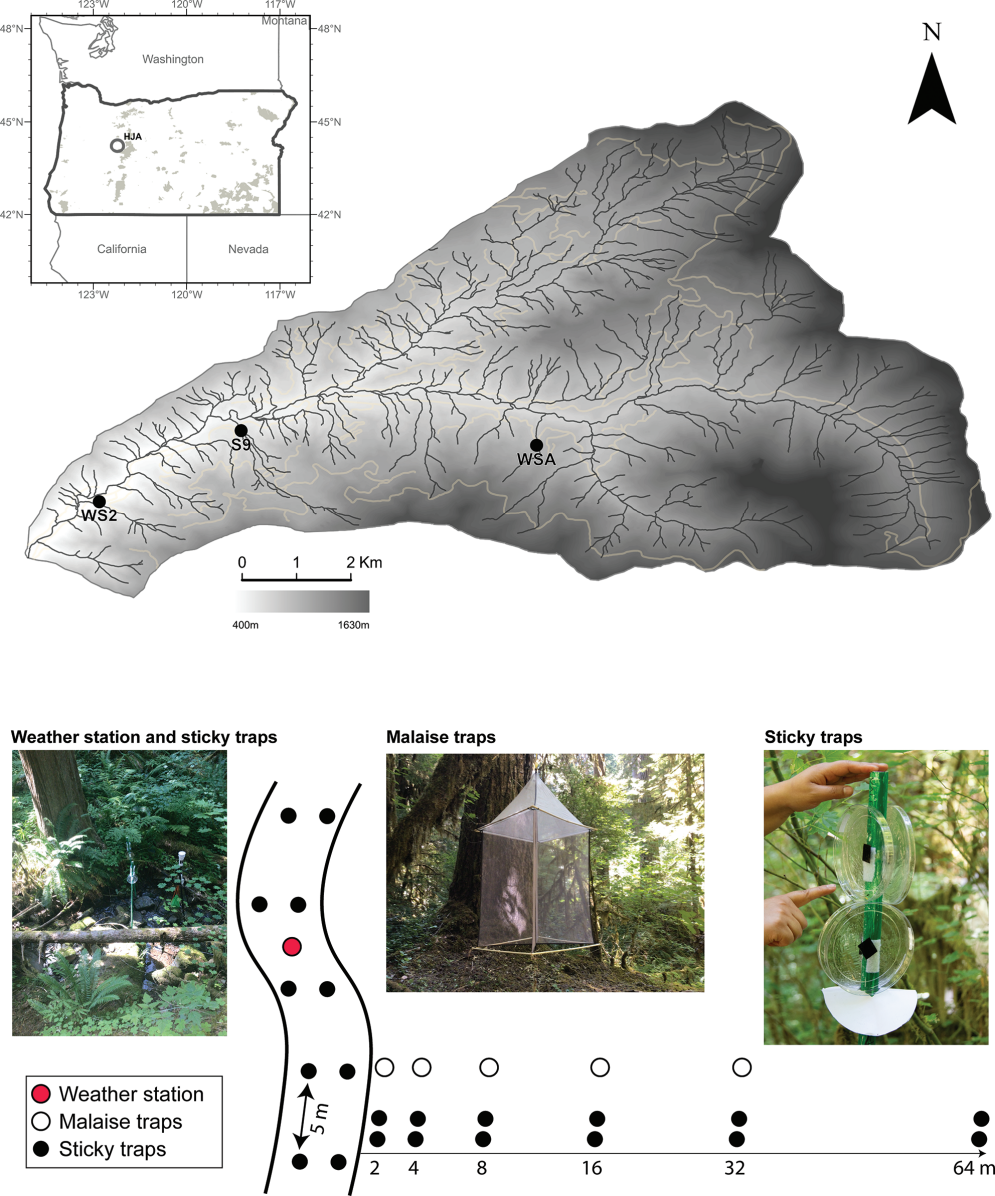
Study map sites and sampling design. On top is a map of the stream network in the Lookout Creek basin, OR with the three sampling sites marked. On the bottom, the sampling design and traps are used to collect insects lateral and longitudinally at WS2.

Our study was conducted in the H.J. Andrews Experimental Forest, Cascade Mountains, Oregon, USA (Fig. 1, top). This long-term ecological research site is contained in a 6400-ha drainage basin with elevations from 410 to 1630 m above sea level (Becker et al. 2023). Riparian trees consist of Douglas fir (*Pseudotsuga menziesii*), western red cedar (*Thuja plicata*), and big-leaf maple (*Acer macrophyllum*) (Frady et al. 2007). Our sampling occurred between July 10 and August 10, 2023. We did not continue our sampling after August 10 due a wildfire that occurred at the H.J. Andrews Experimental Forest (HJ Andrews Experimental Forest 2023).

### ﻿Collection of adult insects

For lateral dispersal, data were collected from three second-order tributaries of Lookout Creek in drainages with forests of differing ages (Table 1, Fig. 1) whereas for longitudinal dispersal we focused our efforts in one of these tributaries (WS2). To describe the lateral dispersal at all three tributaries, we installed PVC posts (150 cm height) from the edge of the stream and further into the riparian zone following a logarithmic-based 2-scale (2, 4, 8, 16, 32, and 64 m) as previous research suggested (Didham et al. 2012). Four circular sticky traps (plastic Petri dishes of 15 cm in diameter) were attached to each PVC post based on the prototype developed by Smith et al. (2014). The Petri dishes faced four directions (upstream, downstream, left side, and right side) and two posts per location were installed as replicates at each tributary. All Petri dishes were collected and preserved weekly for five weeks and taken to the lab to identify and enumerate the captured insects. Identifications were to the finest practical level, typically to family level as the glue from the traps did not allow further examination of taxonomic features. (Fig. 1, bottom, right). Malaise traps (Malaise 1937) were also deployed at WS2 to explore lateral insect dispersal further. Five suspended Malaise traps were set 1 m above ground-level along a lateral transect at distances from the stream following a logarithmic-based 2-scale (2, 4, 8, 16, and 32 m; Fig. 1, bottom-left). The traps were emptied, and the preservation solution of ethanol (90%) was replaced weekly for a total of five weeks. Specimens captured in the Malaise traps were identified to the finest practical level, typically to the genus or species level.

**Table 1.** Site and sampling description of the three study headwater streams of the H.J. Andrews Experimental Forest. Stream width and depth mean correspond to Summer 2022.

Stream	Sampling method (Traps)	Forest age (years)	Coordinates	Altitude (m a.s.l.)	Mean air temperature (°C)	Stream width^†^ and depth^*^ (m)
WS2- Longitudinal	Sticky	Old > 450	44.2147, −122.2494	489	19.4	1.68**^†^** 0.13^*^
WS2- Lateral	Sticky/ Malaise					
S9- Lateral	Sticky	Young (NA)	44.2265, −122.2260	573	19	1.69**^†^** 0.18^*^
WSA-Lateral	Sticky	Young > 50	44.2240, −122.1771	764	18.6	2.52**^†^** 0.20^*^

We assessed longitudinal dispersal in WS2 as a first attempt to test the association between dispersal direction of each taxon and the patterns of local wind (i.e., speed and direction). We used a similar sticky traps deployment described above, but five replicated posts were set along the stream channel located 5 m apart from each other covering 30 m section of the stream channel.

### ﻿Microclimate

For longitudinal dispersal at WS2, we used a Tempest Weather System (https://weatherflow.com/tempest-home-weather-system/) deployed at the middle of a section of the stream channel positioning the designated North indicator of the weather system towards the upstream direction. Climatic variables, including wind speed and direction, were recorded every 5 min during our study period.

### ﻿Data analyses

To test the shape of the dispersal pattern of adult flying insects we pooled the samples and fit a negative exponential function based on the distance from the stream channel. We used this procedure to visualize taxa richness, total abundance, and percentage of aquatic insects for lateral dispersal. We used the nonlinear regression with an exponential decay tool implemented in Sigma Plot v. 15 software (SYSTAT Software, Inc. 2022). For the longitudinal dispersal, in addition to the adult aquatic insect identification and count, the Trainable Weka Segmentation protocol (Arganda-Carreras et al. 2017) inside ImageJ and ImageJ macros were used to automate the arthropod count and size process (Suppl. material 1). Regarding the microclimate variables, Circular statistics package in R (Agostinelli and Lund 2024) were used for the analysis and visualization of wind direction and speed.

## ﻿Results

### ﻿Lateral dispersal

During the five weeks, more than 4800 flying insects (terrestrial and aquatic) were collected using the sticky trap method in WS2, S9, and WSA tributaries. Diptera (42.3%), Coleoptera (26.6%), and Hymenoptera (18.2%) were the most abundant terrestrial orders. Regarding the aquatic orders, Diptera (59.4%) and Plecoptera (19%) represented the majority, while Trichoptera and Ephemeroptera were 13.5% and 8.1%, respectively. Using the Malaise traps in WS2, more than 2300 insects were collected. Diptera (55.7%), Lepidoptera (11.3%), and Coleoptera (10.47%) were the most abundant terrestrial orders, while Trichoptera (33.6%) and Ephemeroptera (30.2%) dominated the aquatic orders.

**Figure 2.**
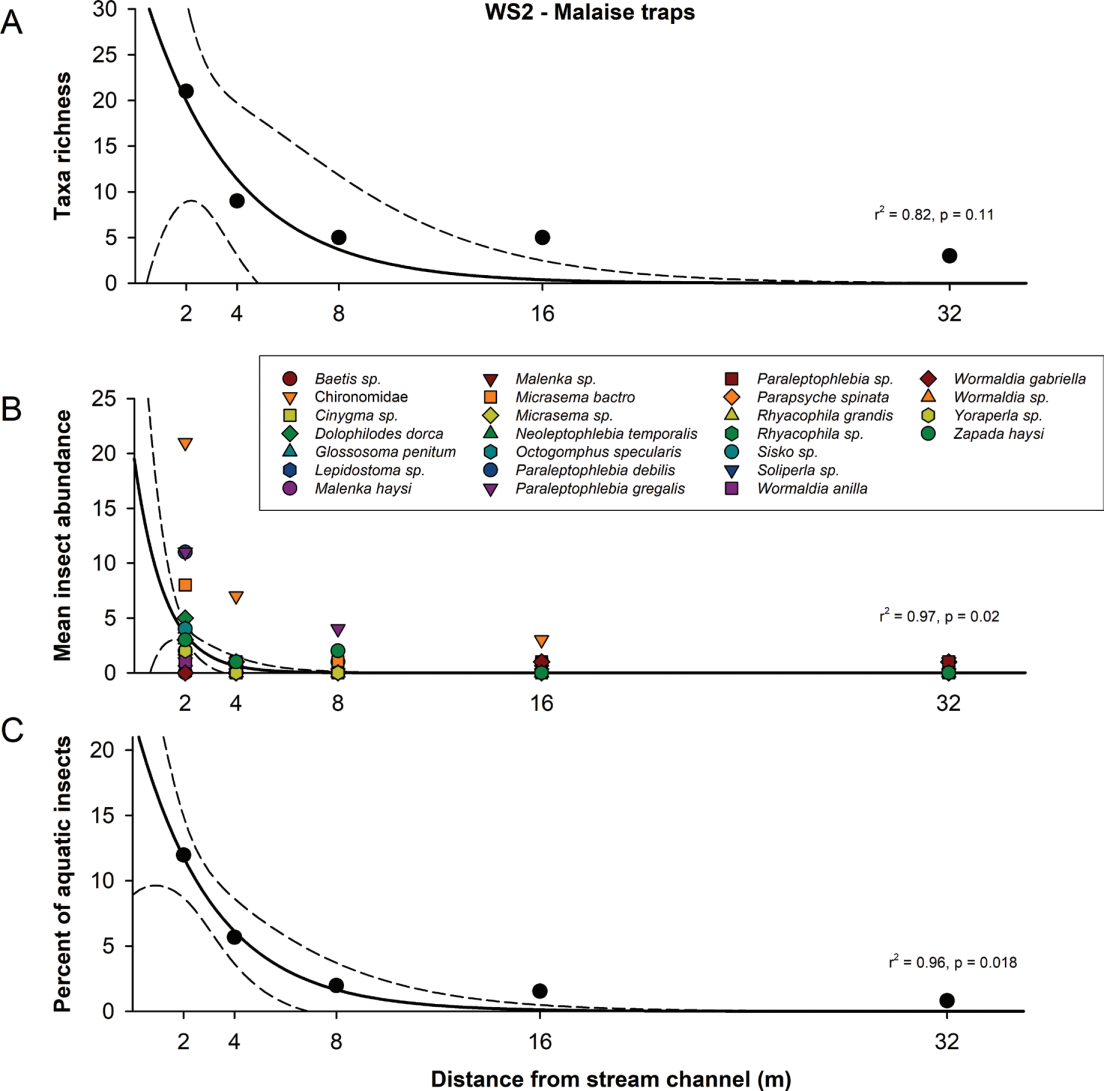
Exponential decay of Ephemeroptera, Plecoptera, Trichoptera (EPT) and Chironomidae for Malaise traps. Nonlinear regression with exponential decay (f _(x)_ = *a*e^−^*^bx^*) of EPT genera/species in Malaise traps set in five different distances at WS2 during five trials. **A.** Total of EPT genera/species and Chironomidae; **B.** Mean abundance of EPT genera/species and Chironomidae**C** percentage of aquatic insects per trap. The solid line represents the nonlinear regression, and the dashed line represents the 95% confidence interval.

**Figure 3.**
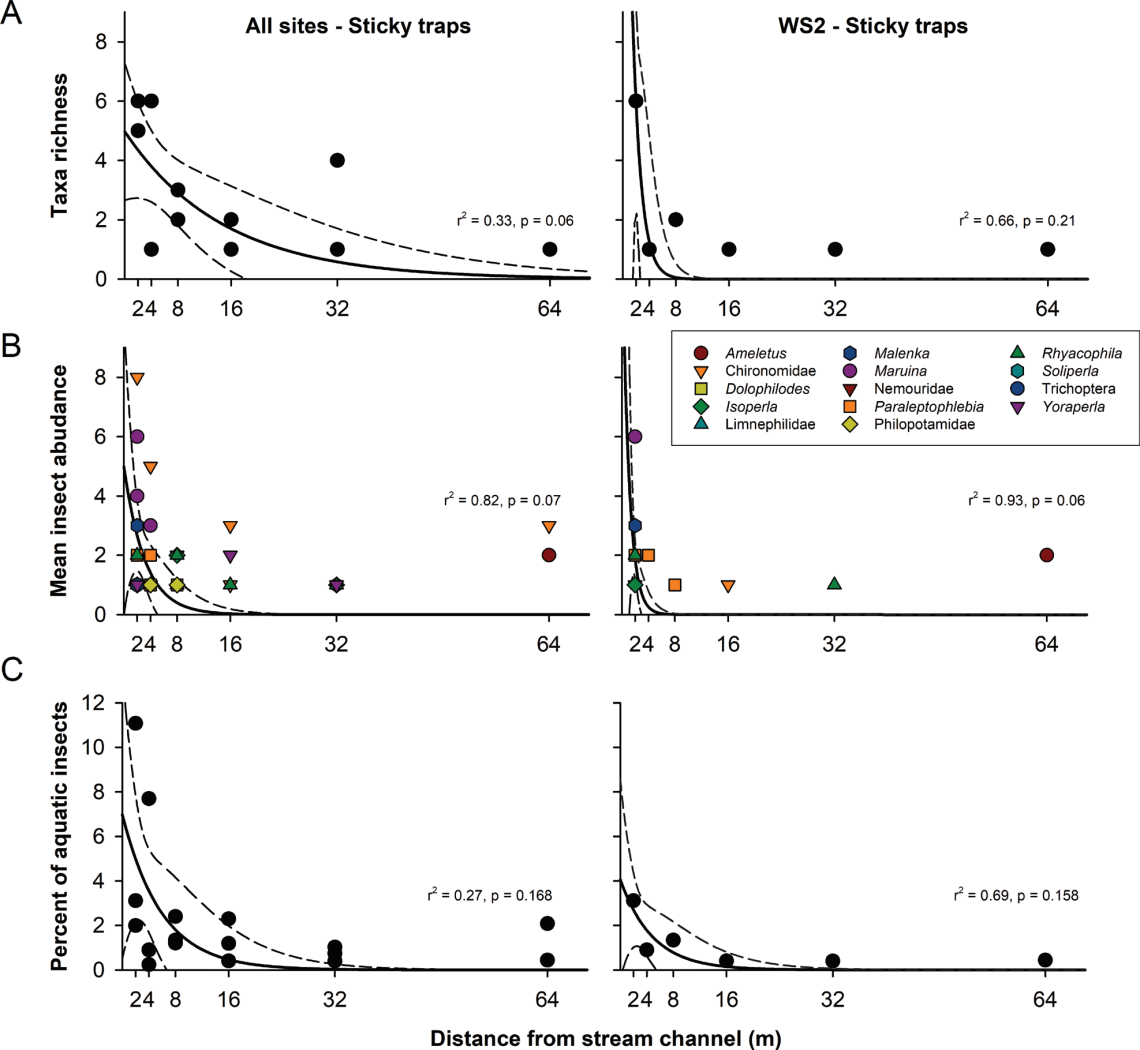
Exponential decay of EPT and Chironomidae for sticky traps. Nonlinear regression with exponential decay (f _(x)_ = *a*e^−^*^bx^*) of EPT family/genera in sticky traps set in six different distances at all sites (left column) and WS2 (right column) during five trials. **A** Number of families/genera; **B** Mean of number of individuals per taxa; **C** Percentage of aquatic insects per trap. The solid line represents the nonlinear regression, and the dashed line represents the 95% confidence interval.

Nonlinear regression with exponential decay analysis showed significant differences (*p* < 0.05) between distances for species mean abundance (Fig. 2B) and percent of aquatic insects (Fig. 2C) when using Malaise traps. No differences were observed between families-genera found in sticky traps for all tributaries or WS2 (Fig. 3).

### ﻿Longitudinal dispersal

**Figure 4.**
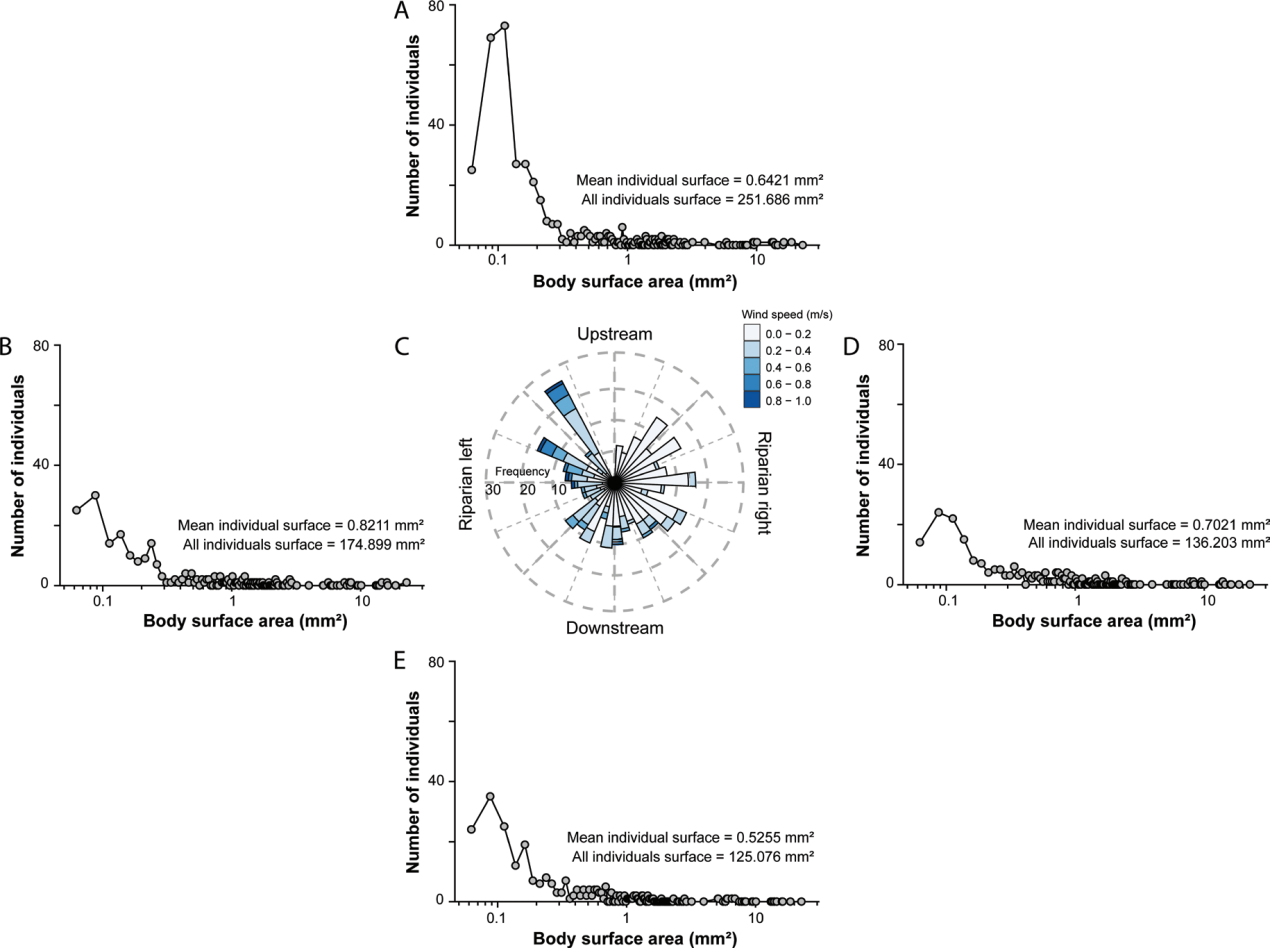
Automated count and size of arthropods for longitudinal sampling using the Trainable Weka Segmentation protocol. Each graph corresponds to the cumulative number of insects (aquatic and terrestrial) found in the Petri dish per direction. **A** Upstream; **B** Left side; **C** Wind direction and speed during the five weeks; **D** Right side; **E** Downstream.

**Figure 5.**
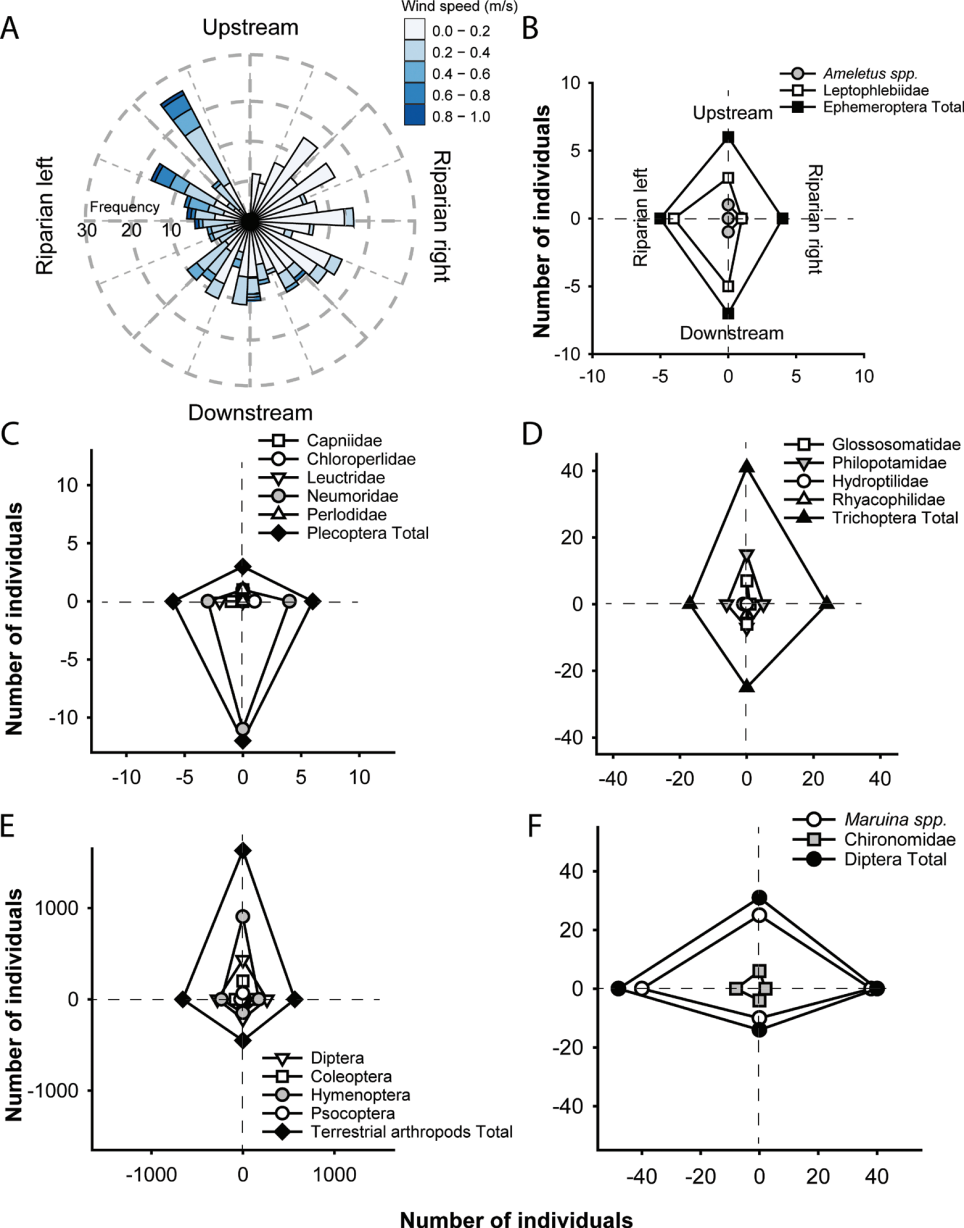
Patterns of wind speed and direction and longitudinal dispersal. For EPT and aquatic Diptera abundance. **A** Wind direction and speed during the five trials; **B**Ephemeroptera and families; **C**Plecoptera and families; **D**Trichoptera and families; **E** terrestrial arthropods and orders; **F** aquatic Diptera.

Most arthropods collected were small (less than 0.5 mm^2^ of body surface area, Fig. 4). Also, arthropods tended to disperse in greater numbers in the same direction as the wind, in the upstream direction. Regarding aquatic insects, more than 150 adults were collected during the study period. When comparing the direction in which the aquatic orders disperse, different patterns of dispersal were observed for the more abundant families. Leptophlebiidae (Ephemeroptera, Fig. 5B) and Diptera (Fig. 5F) dispersed mainly laterally, Nemouridae (Plecoptera, Fig. 5C) dispersed mainly downstream, and Philopotamidae (Trichoptera, Fig. 5D) dispersed mainly upstream.

### ﻿Notes about Trichoptera

Caddisflies were the most diverse aquatic order for lateral and longitudinal dispersal. Six Trichoptera families were collected including Brachycentridae, Glossosomatidae, Philopotamidae, Rhyacophilidae, Limnephilidae, and Lepidostomatidae. Four and three families were found in the longitudinal and lateral sticky traps, respectively. A total of 11 species were identified from the Malaise traps (Fig. 2B) and most of them (10 species) were found closer to the stream channel (2 m trap). *Micrasema bactro* (Brachycentridae), one of the smallest species found (Table 2), was consistently collected across distances, even in the Malaise trap located 32 m further away from the tributary.

**Table 2.** Sex percentage and mean size (mm) of EPT genera/species in Malaise traps during the five trials.

Genera/ Species	Number of individuals	Female proportion	Female mean size ± SD (mm^2^)	Mean size (mm^2^)
** Ephemeroptera **				
*Baetis* sp.	2	0.5	3.90	5.29
*Cinygma* sp.	1	0.0		9.25
*Neoleptophlebia temporalis*	3	0.0		6.07 ± 0.2
*Paraleptophlebia debilis*	11	0.1	6.07	6.75 ± 0.79
*Paraleptophlebia gregalis*	17	0.4	5.72 ± 1.35	6.19 ± 0.52
*Paraleptophlebia* sp.	2	1.0	5.70 ± 0.71	
** Plecoptera **				
*Malenka* sp.	1	1.0	6.50	
*Soliperla* sp.	1	1.0	9.91	
*Yoraperla* sp.	2	1.0	5.4 ± 0.42	
*Zapada haysi*	6	0.0		4.62 ± 0.44
** Trichoptera **				
*Dolophilodes dorca*	6	0.5	4.82 ± 0.27	5.01 ± 0.31
*Glossosoma penitum*	1	0.0		5.20
*Lepidostoma* sp.	2	1.0	5.41 ± 0.2	
*Micrasema bactro*	11	0.5	4.02 ± 0.32	3.59 ± 0.14
*Micrasema* sp.	2	1.0	3.55 ± 0.11	
*Parapsyche spinata*	3	0.0		8.90 ± 0.44
*Rhyacophila grandis*	5	0.8	11.54 ± 0.49	9.24
*Rhyacophila* sp.	1	1.0	10.50	
*Sisko* sp.	5	0.2	3.90	3.43
*Wormaldia anilla*	2	1.0	4.27 ± 0.19	
*Wormaldia gabriella*	1	0.0		5.40
*Wormaldia* sp.	3	1.0	3.53 ± 0.25	

## ﻿Discussion

This study evaluates lateral and longitudinal dispersal of adult aquatic flying insects using sticky traps at three small mountain streams and Malaise traps in one of the streams. As noted in our hypothesis for lateral dispersal, most of the aquatic insects are captured closer to stream channels for both Malaise and sticky traps. The results of Malaise traps show that the lateral dispersal of all aquatic insect species collected in a transect of 32 m fit the negative exponential decay curve, suggesting a random dispersal (Rees 1993). However, no differences are observed among distances using sticky traps by each of the families and genera. This could be due to the level of identification achieved, since most of the specimens collected in the sticky traps were damaged during removal for identification.

The species we collected during our study period coincide with phenological observations from previous studies on Leptophlebiidae, Ephemeroptera (Lehmkuhl and Anderson 1971; Dieterich and Anderson 1995; Finn et al. 2022). Although not the most abundant, common species for the region include the mayfly *Neoleptophlebia temporalis* (McDunnough, 1926). *N. temporalis* is expected to emerge from April to June at lower elevations. *Paraleptophlebia debilis* (Walker, 1853), also present close to the stream edge, is considered as a later emerging species. In addition, *Paraleptophlebia gregalis* (Eaton 1883), disperse along the sampled transect up to 32 m. In a study including temporary streams, *P. gregalis* emerged until September with a peak in June.

### ﻿Notes about Trichoptera

Caddisflies are the most diverse order in comparison to Ephemeroptera and Plecoptera in our study. Philopotamidae, the most diverse family, is represented by three genera, and four species. The caddisfly *Dolophilodes dorca* (Ross, 1938) (Trichoptera, Philopotamidae) is a common species for the region that emerges in May/June and is less common in July/August (Anderson et al. 1984; Farrand 2004). In addition to two species of *Wormaldia* (e.g., *W. gabriella* and *W. anilla*), we also collected specimens of the genus *Sisko*, whose taxonomy has been changing in the last years and its larval stages have been recently described (Lee 2024).

While most caddisflies remain close to the stream edge in our study streams, the species *M. bactro*, a less common species in this study with a mean size of 3.6mm, is present along the transect up to 32 m. Some studies have documented caddisfly dispersal distances positively related to insect size, for example, in Limnephilids (Sode and Wiberg‐Larsen 1993; Petersen et al. 1999). However, more recent studies related to traits or metrics such as body or wing size have shown a lack of empirical evidence to draw these conclusions (Lancaster et al. 2024a).

### ﻿Longitudinal dispersal and microclimate influence

We evaluate a procedure as proof of concept to measure longitudinal dispersal using sticky traps in one of our study sites. We show evidence that supports the hypothesis of longitudinal dispersal influenced by the magnitude and direction of winds. Indeed, we show longitudinal dispersal consistent among small and most abundant arthropods (aquatic and terrestrial) that coincide with the direction of the wind. In this case, sticky traps are more informative compared to Malaise traps regarding directions when the research questions include microclimate variables such as wind direction, as in this study.

Our findings align with other studies that consider upstream flight as evidence for the colonization cycle hypothesis (Müller 1982). However, when analyzing the EPT orders and the most abundant Diptera family in our dataset, the movement of each group tends to be different. Plecoptera, for example, appears to move downstream, in contrast to another study in which the species *Alloperla ishikariana* had a greater number of individuals moving upstream (Rahman et al. 2021). This could be due to species-specific flight dispersal preference.

Although we do not reach the level of species identification for longitudinal dispersal, the families we observe can disperse in different directions. However, it is necessary to implement more adequate capture methods that allow a better association between wind and each species. An alternative is the use of double-headed Malaise trap with a closed median partition and different alcohol containers to capturing specimens on each side to test directional dispersal (Briers et al. 2003).

### ﻿Limitations and next steps

Our study contributes to the empirical evidence of the dispersal of aquatic insects once they emerge and contributes to the ecological and biological knowledge of some common genera in the region. However, the low density of insects captured, the number of sites compared, and the relatively short sampling time limit strength of our conclusions about the dispersal patterns of the aquatic orders. We recommend future sampling be carried out to include a more extended time window, biomass measurements of both aquatic and terrestrial groups, and a larger number of sampling sites. Finally, this work constitutes the baseline for investigating the effects of disturbances such as wildfires or forest harvest and their effect on the aquatic communities that link the streams to the riparian zone.

## ﻿Conclusion

Our study provides new empirical evidence on how adult aquatic insects bridge stream‐riparian boundaries in headwater forests of the H.J. Andrews Experimental Forest. First, lateral dispersal measured with Malaise traps conformed to a clear negative-exponential decay: > 70% of individuals were captured within the first 8–16 m from the channel, confirming that most emerging EPT taxa remain tightly coupled to the stream corridor. Nonetheless, the consistent capture of *Micrasema bactro* up to 32 m demonstrates that even small caddisflies can exceed commonly assumed dispersal limits, underscoring the need to consider species-specific traits when delineating riparian buffer widths. Second, our proof-of-concept assessment of longitudinal dispersal showed that total arthropod flux—and the upstream bias of Philopotamidae in particular—closely tracked prevailing wind direction and speed, highlighting microclimate as an overlooked driver of along-stream dispersal. In contrast, Nemouridae tended to move downstream, while Leptophlebiidae and most Diptera dispersed laterally, illustrating that families adopt distinct strategies rather than a single, taxon-wide “colonization cycle.”

Together, these results (i) establish a simple, low-cost framework that couples sticky traps with on-site weather monitoring to capture multi-directional dispersal; (ii) furnish baseline distance functions that can be re-measured after wildfires, timber harvest, or climate shifts to detect change; and (iii) emphasize that conserving microclimatic heterogeneity in riparian zones is as critical as retaining forest cover itself. Future work that extends sampling through the full emergence season, integrates biomass and species-level identifications, and employs bidirectional Malaise traps will be essential for refining these insights and for forecasting how disturbance regimes reshape aquatic–terrestrial connectivity in forested watersheds.

## ﻿Additional information

### Author ORCIDs

Tatiana Latorre-Beltrán https://orcid.org/0000-0002-7972-0160

Ivan Arismendi https://orcid.org/0000-0002-8774-9350

Blanca Rios-Touma https://orcid.org/0000-0002-3921-0908

William Joseph Gerth https://orcid.org/0000-0001-6050-1550

Alexander Petty https://orcid.org/0009-0007-4165-6873

### Data availability

All of the data that support the findings of this study are available in the main text or Supplementary Information.

## Supplementary materials

Supplementary material 1 Supplementary material guide Data type docx https://binary.pensoft.net/file/1487870This dataset is made available under the Open Database License (http://opendatacommons.org/licenses/odbl/1.0/). The Open Database License (ODbL) is a license agreement intended to allow users to freely share, modify, and use this Dataset while maintaining this same freedom for others, provided that the original source and author(s) are credited.Tatiana Latorre-Beltrán, Ivan Arismendi, Blanca Rios-Touma, William Joseph Gerth, Alexander Petty

Supplementary material 2 PetriDishIdentifier Data type ijm https://binary.pensoft.net/file/1487871This dataset is made available under the Open Database License (http://opendatacommons.org/licenses/odbl/1.0/). The Open Database License (ODbL) is a license agreement intended to allow users to freely share, modify, and use this Dataset while maintaining this same freedom for others, provided that the original source and author(s) are credited.Tatiana Latorre-Beltrán, Ivan Arismendi, Blanca Rios-Touma, William Joseph Gerth, Alexander Petty

Supplementary material 3 InsectIdentifierMacro Data type ijm https://binary.pensoft.net/file/1487872This dataset is made available under the Open Database License (http://opendatacommons.org/licenses/odbl/1.0/). The Open Database License (ODbL) is a license agreement intended to allow users to freely share, modify, and use this Dataset while maintaining this same freedom for others, provided that the original source and author(s) are credited.Tatiana Latorre-Beltrán, Ivan Arismendi, Blanca Rios-Touma, William Joseph Gerth, Alexander Petty
