# Supplementary material for: ﻿Lateral and longitudinal dispersal of aquatic insects in mountain streams, with notes about Trichoptera
Source: Zookeys. 2025 Dec 10;1263:317–31. doi: 10.3897/zookeys.1263.150229 (PMC12712621; doi:10.3897/zookeys.1263.150229)
Supplement: Supplementary material 1 — Supplementary material guide [file zookeys-1263-317_article-150229__-s001.docx]

**Supplementary Material Guide**

This supplemental material will go into more detail on automating insect count and size from images of Petri dishes.

**The main tools used:**

- **ImageJ:**  An image-processing library mainly for scientific applications. Version ImageJ1.54f used.
- **ImageJ macros:** Can automate any action manually done in ImageJ. Comes automatically with ImageJ. Used for running many actions on a folder of images.
- **Hough Circle Transform:** A plugin for ImageJ used in the petri dish identifier macro used to identify circles. Needs to be installed inside ImageJ.
- **Trainable Weka Segmentation:** A plugin for ImageJ that allows for training a neural network to segment an image. In our case using it to separate insects from the background. Version 3.3.4 used.

**The Process**

Starting with a folder of images of petri dishes, an example of one is shown in Figure 1:


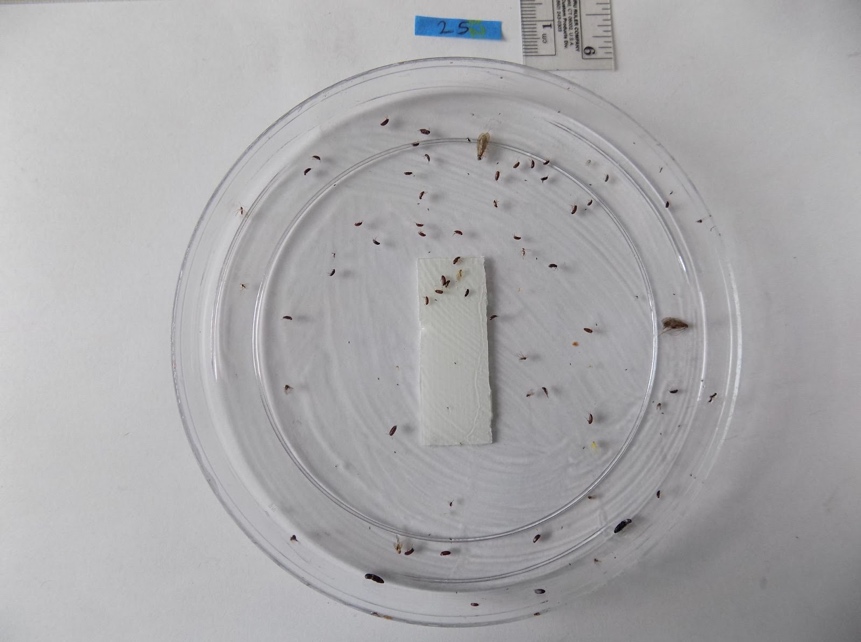


Fig 1. Sample Photo

**Identifying the Petri Dish**

We first need to make the petri dish take up the whole image shown in Figure 2. This eliminates background noise making future steps easier. Secondly, due to the petri dish taking up the entire image width, we now have the petri dish’s width in pixels. Then, by measuring the width in real life, we have a way to convert between pixels and useful units such as millimeters. This is done by running an ImageJ macro on the folder of petri dish images outputting a folder of image with only petri dish. While the actual conversion of units happens when running the insect identifier macro in a future step. The petri dish identifier macro can be found under “2. PetriDishIdentifier.ijm”.


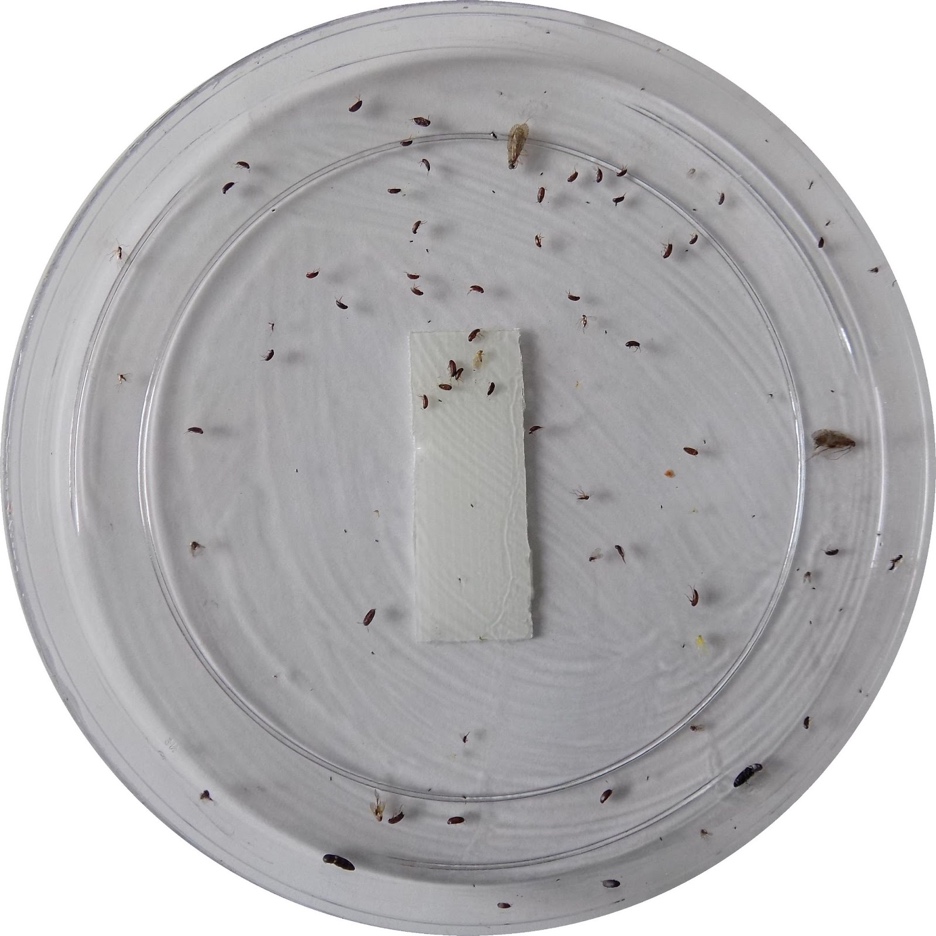


Fig 2. Only Petri Dish

**Training the Classifiers**

Once we have only the petri dish image, we can use Weka Segmentation to identify the insects. This is done by training a classifier with a GUI interface, shown in Figure 3. Then, run the classifier on any image we want to output a black and white image of what it identified as insects and what it identified as background.

When training we found it easier to train two classifiers, one for moths and another for smaller insects, and then combine the results. Training one classifier either led to misidentifications of shadows on smaller insects due to faded moth-wing-looking shadows or fragmentation of moth wings when trying to account for the shadows.


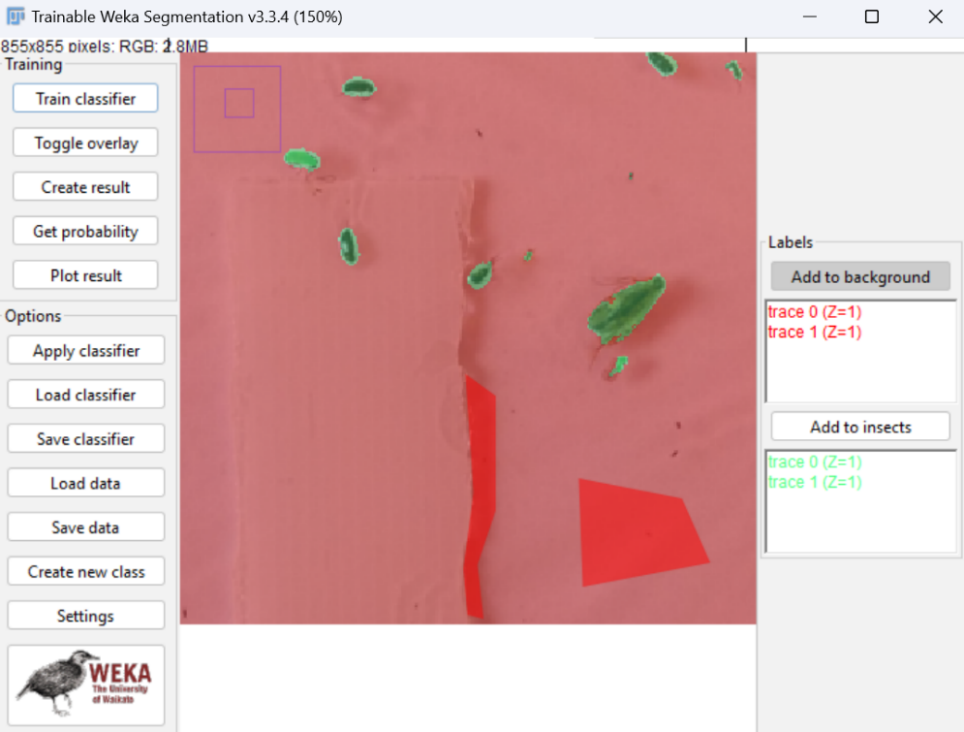


Fig 3. Trainable Weka Segmentation GUI

**Running the “Identify Insect” Macro**

Once the classifiers were trained, we used an ImageJ macro to run the classifiers on the folder of petri dish images. The macro can be found under “3. IdentifyPetriDish.ijm”.

We will go over the process for one image in the folder.

1. The macro will first run Trainable Weka Segmentation with the insect classifier on a petri dish shown in Figure 2, along with filtering out any areas below 0.4mm^2^ in size to reduce misidentifications of small specks. This produces a black-and-white image shown in Figure 4.


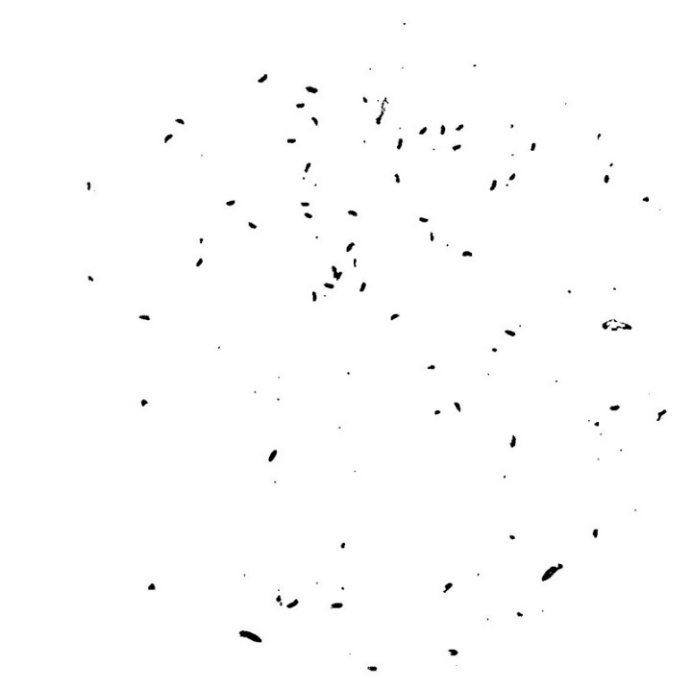


Fig 4. Identified Insects from Figure 2

2. The macro will output a black-and-white image of just the larger insects and moths using the moth classifier and filtering out any areas below 5mm^2, shown in figure 5.


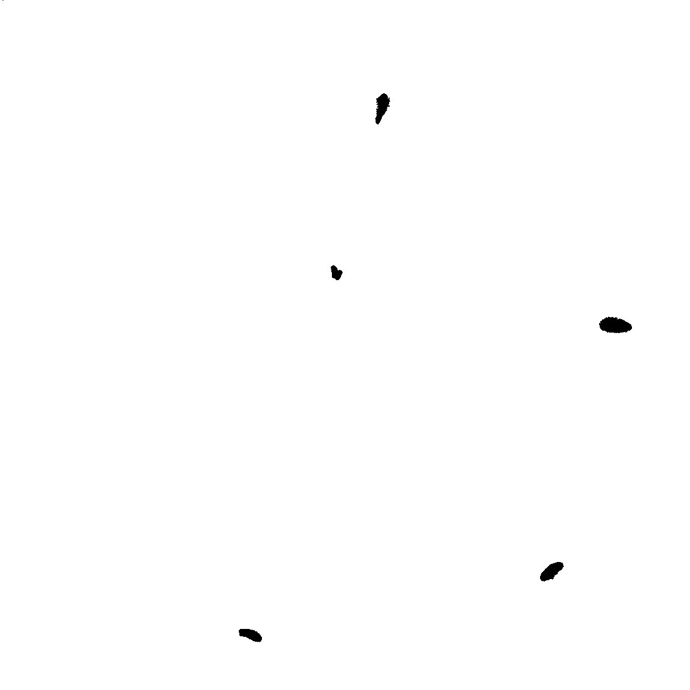


Fig 5. Identified Moths from Figure 2

3. Both of these images are then combined as shown in Figure 6


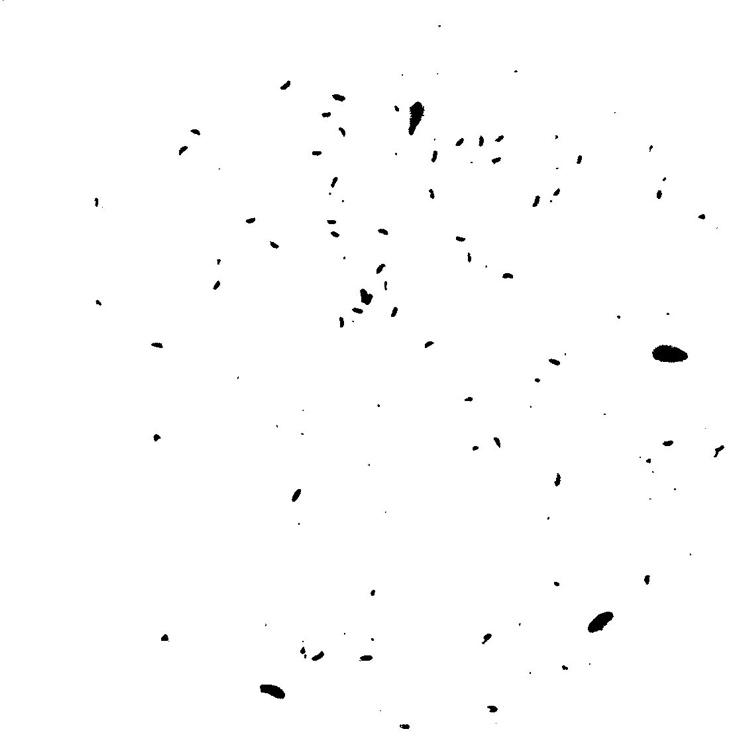


Fig 6. Combined Moth and Insects

4. ImageJ’s analysis particles is then ran on the combined image. Analysis particles counts the size of groups of pixels from a black-and-white image. This outputs a result CSV (comma-separated values) containing the size of every insect identified shown in Figure 7 and a summary CSV containing the count and average size of insects from each image shown in Figure 8.


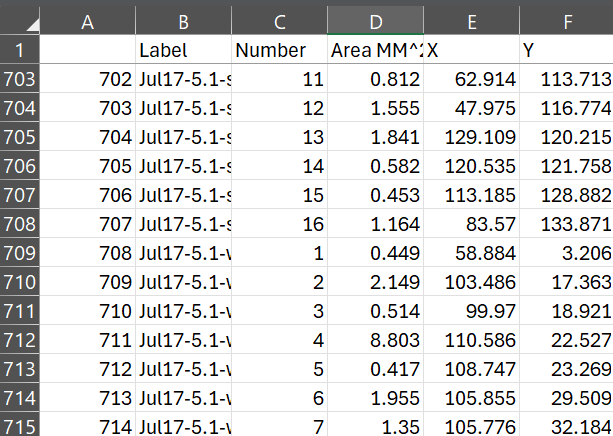


Fig 7. Section of Result CSV


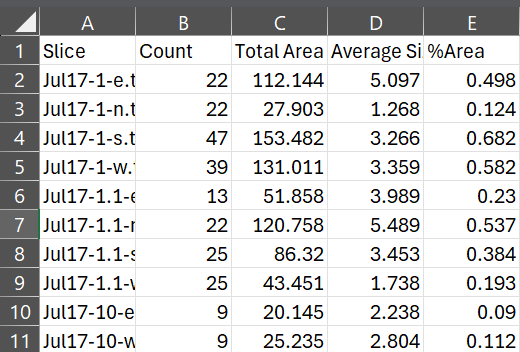


Fig 8. Section of Summary CSV

5. The detected insects are then overlaid onto the original image, along with numbering each overlay for error checking shown in Figure 9 and Figure 10. Each number will correspond to a box in the ID column of the result CSV allowing for tracking down specific insects or correcting misidentifications in the CSV. For example, some of our images have black squares that the classifier detected as insects, we were able to find and remove them from the CSV.


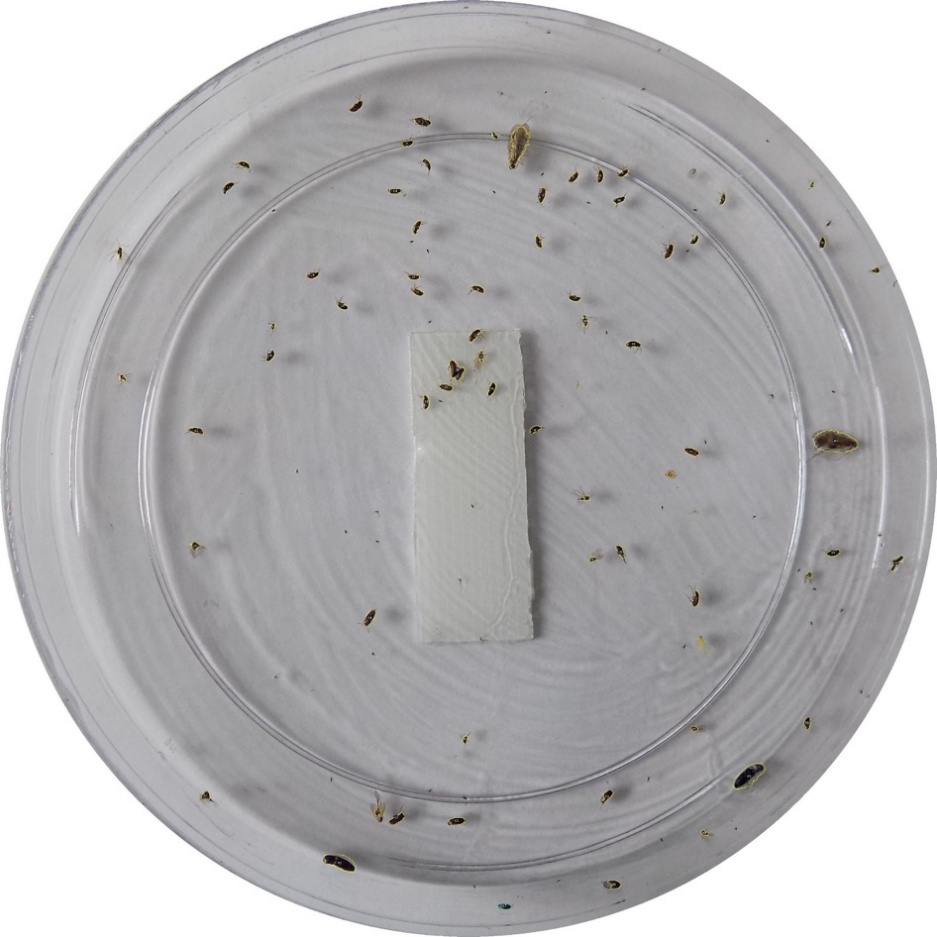


Fig 9. Identified Insects overlaid on Petri dish


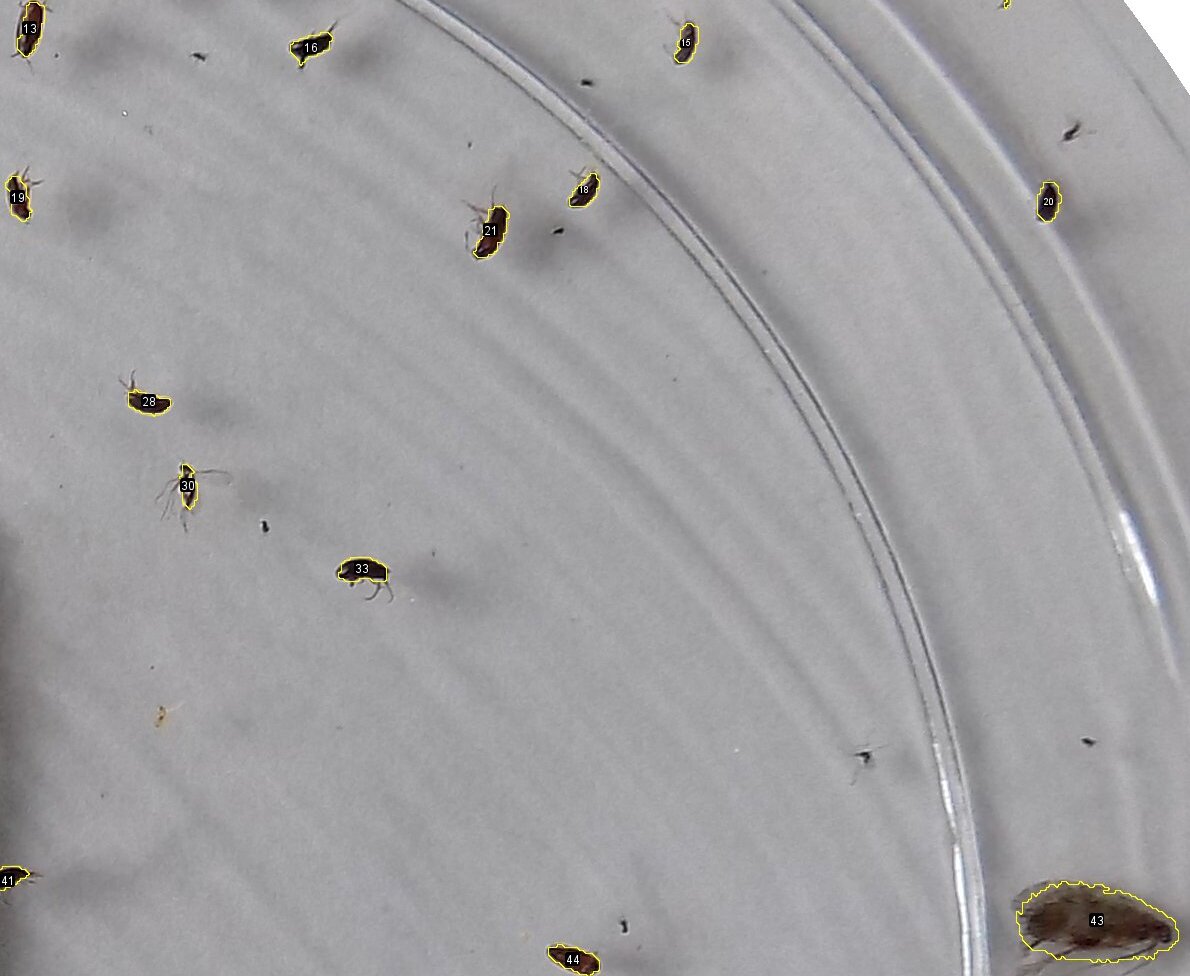


Fig 10. Zoom in on the overlay
